# Dataset on evolution analysis of splenic transcriptome in bighead carp and silver carp

**DOI:** 10.1016/j.dib.2019.01.003

**Published:** 2019-01-05

**Authors:** Guoxi Li, Yinli Zhao, Shuang Guo, Bianzhi Liu, Yi Chen, Xiangli Sun, Jianxin Feng

**Affiliations:** a College of Animal Science and Veterinary Medicine, Henan Agricultural University, Zheng zhou, Henan Province 450002, PR China; b College of Biological Engineering, Henan University of Technology, Zheng zhou, Henan Province 450001, PR China; c Laboratory of Aquaculture and genetic breeding, Henan Academy of Fishery Science, Zheng zhou, Henan Province 450044, PR China

## Abstract

Bighead carp (*Aristichthys nobilis*) and silver carp (*Hypophthalmichthys molitrix*) are closely related species in the subfamily *Xenocypridinae* within *Cyprinidae*, and they are also two of the four most important pond-cultured fish species in China. The ability to resist some diseases often differs significantly in silver carp and bighead carp during fishery production. However, the evolutionary divergence of the immune defense functions in these two species is still not understood at the molecular level. The data presented in this article are related to the research article entitled “Comparative analysis of spleen transcriptome detects differences in evolutionary adaptation of immune defense functions in bighead carp and silver carp” (Li et al., 2018). Please refer to this data article for interpretation of the data. Data provided in this submission comprise the Ka/Ks ratios of orthologs as well as adaptive evolution genes, expression levels of orthologs, and TPM value of genes expressed only in spleen of bighead carp or silver carp. These data provide a better understanding of the differences in evolutionary adaptation of immune defense functions in bighead carp and silver carp.

**Specifications table**

**Table:** 

Subject area	Biology
More specific subject area	Transcriptomics
Type of data	Excel file and word table
How data were acquired	High-throughput RNA-sequencing
Data format	Analyzed
Experimental factors	All animal experiments were performed as described previously [1]. The spleen samples were collected from three-year-old bighead carp (*A. nobilis*) and silver carp (*H. molitrix*).
Experimental features	cDNA libraries were constructed using a Truseq^TM^ RNA sample prep Kit (Illumina). The paired-end cDNA library was sequenced on an Illumina HiSeq. 4000. Evolution analysis and expression analysis of genes were performed as described in [1]. The estimate of Ka/Ks values were performed using the CodeML program in the PAML package [2].
Data source location	Yellow River basin in Xingyang, Henan Province, China
Data accessibility	Analyzed datasets are contained within this article. Raw data were deposited in the NCBI database Sequence Read Archive. The accession numbers for the bighead carp and silver carp sequences are SRP078549 and SRP078474, respectively.

**Value of the data**

• The data provide the results of evolutionary analysis of orthologs in spleen between bighead carp and silver carp, which is an important basis for comparative genomic studies of adaptation in silver carp, bighead carp, and other closely related species, as well as for understanding the genetic basis of differences in resistance to some diseases in silver carp and bighead carp.
• The data also provide the results of gene expression analysis in spleen of bighead carp and silver carp, which contributes to the understanding of transcriptome profiling of spleen tissue in silver carp and bighead carp.

## 1 Data

The transcriptome sequencing from the spleen tissue of silver carp and bighead carp was performed using Illumina paired-end sequencing technology. The dataset of this data article comprises seven data files that were generated from the evolutionary analysis of orthologs and gene expression analysis of the transcriptome sequence above. The orthologs as well as their information of Ka/Ks ratios are presented in File 1. The Ka/Ks ratios of adaptive evolution genes are presented in File 2. The expression levels of orthologs are presented in File 3. The genes expressed only in spleen of bighead carp or silver carp are presented in File 4 and File 5, respectively. Disease pathways assigned based on the KEGG databases are presented in File 6. Adaptive evolution of four genes as markers for spleen development is shown in File 7.

## 2 Experimental design, materials and methods

### 2.1 Experimental animals

The experimental animals comprised bighead carp and silver carp collected from the Yellow River basin in Xingyang, Henan Province, China. The spleen tissues were collected from six healthy individuals per each species.

### 2.2 cDNA library construction and sequencing

The cDNA library was constructed by using Truseq^TM^ RNA sample prep Kit (Illumina) and sequenced on an Illumina HiSeq. 4000 sequencing platform that generated paired-end reads of 151 nucleotides. The detailed description can be seen in Ref. [1].

### 2.3 Determination of orthologs

Orthologs among the bighead carp and silver carp were determined using OrthoMCL [3]. The detailed description can be seen in Ref. [1].

### 2.4 Substitution rate estimation for adaptive genes

The CodeML program in the PAML package was used to estimate the ratio (Ka/Ks values) of the number of non-synonymous substitutions per non-synonymous site (Ka) relative to the number of synonymous substitutions per synonymous site (Ks) [2].

### 2.5 Gene expression analysis

Gene expression levels were calculated using the transcripts per million (TPM) method. The detailed description can be seen in Ref. [1].
