# Supplementary material for: Dataset on evolution analysis of splenic transcriptome in bighead carp and silver carp
Source: Data Brief. 2019 Jan 5;22:812–4. doi: 10.1016/j.dib.2019.01.003 (PMC6348730; doi:10.1016/j.dib.2019.01.003)
Supplement: Supplementary file 1 — Supplementary material [file mmc1.doc]

Conflict of Interest Form

We certify that all authors have participated sufficiently in the work to take public responsibility for the appropriateness of the experimental design and method, and the collection, analysis, and interpretation of the data. The authors declare that they have no competing interests.
